# Involvement of 5‐HT‐BDNF signaling axis in mediating synergistic antidepressant‐like effects after combined administration of two oligosaccharide esters

**DOI:** 10.1002/fsn3.2098

**Published:** 2021-01-04

**Authors:** Wen Shan Yang, Zhen‐Guo Shi, Xian‐Zhe Dong, Ping Liu, Meng‐li Chen, Yuan Hu

**Affiliations:** ^1^ Department of Pharmacy Medical Supplier Center Chinese PLA General Hospital Beijing China; ^2^ Medical School of Chinese PLA Chinese PLA General Hospital Beijing China; ^3^ Department of Outpatient Group 82 Military Hospital Baoding China; ^4^ Medical Affair Pharmacy Office Chinese PLA General Hospital Beijing China; ^5^ Department of Pharmacy Xuanwu Hospital of Capital Medical University Beijing China

**Keywords:** BDNF, herb activity compounds, multi‐target, synergistic mechanism

## Abstract

Potential mechanisms of depression involving herbal medicines and their specific compounds include elevated 5‐HT level and downstream BDNF pathway. To identify potentially new combined therapeutic strategies, 3,6'‐disinapoylsucrose (DISS) and tenuifoliside A (TFSA) have been observed to show antidepressant‐like effects and its related 5‐HT‐BDNF pathway. We have tried to investigate whether combined administration of DISS and TFSA exerted more effective in the treatment of depression, as assessed through tail suspension test (TST) and forced swimming test (FST). In addition, we also analyzed the expression of three important proteins, cyclic adenosine monophosphate (cAMP) response element binding (CREB), brain‐derived neurotrophic factor (BDNF), and cAMP‐regulated transcriptional coactivators (CRTC1), which have been shown to be involved in the regulation of the neurotrophic factors in the hippocampus. The DISS and TFSA separately, both at a dose of 5 mg/kg each, displayed small effect in the immobility time. However, combined treatment of these two in multiple doses exhibited better effect. Moreover, combined treatment of DISS and TFSA also demonstrated enhanced levels of 5‐hydroxytryptamine (5‐HT), and stronger increase in the phosphorylation levels of CREB, BDNF, and CRTC1 proteins in the hippocampus. Overall, our results indicated that coadministration of these two oligosaccharide esters at low dose may induce more pronounced antidepressant activity, in comparison with individual treatment even at high dosage. Thus, the antidepressant properties of both these compounds can be attributed to their ability to influence 5‐HT and BDNF pathway, and thereby suggesting that this combination strategy can definitely act as alternative therapy for depression disorder with very limited side effects.

## Graphical abstract

The antidepressant properties of DISS and TFSA can be attributed to their ability to influence 5‐HT and BDNF pathway, and thereby suggesting that this combination strategy can definitely act as alternative therapy for depression disorder with very limited side effects.

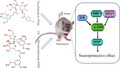

## 1 INTRODUCTION

A growing number of animal studies have demonstrated that underlying mechanism involved in the antidepressant properties of herbal medicines and their specific compounds include elevated levels of 5‐HT and BDNF (An et al., 2008; Hu, Liao, et al., 2010; Hu et al., 2009; Li et al., 2015). In addition, several other studies have indicated rapid enhancement of BDNF expression and synaptic plasticity in the special regions of brain, which is responsible for the antidepressant effect (Udina et al., 2016). Also, the transcription activator CREB has also been shown to be central in response to many signal cascades activated through growth factor, synaptic activity, and other cellular stimuli related to neuronal plasticity (Escoubas et al., 2017; H. Liu et al., 2015; Ning et al., 2019). Moreover, an equally important coactivator CRTC, also known as transducer of regulated CREB (TORC), has been recognized to play an important role in influencing CREB function (Heinrich et al., 2013). More specifically, after CRTC1 translocation from cytoplasm to the nucleus of cortical neurons, CRTC associate with basic leucine zipper domain of CREB independent of its phosphorylation status and subsequently increase CREB transcriptional activity (Liang et al., 2016; Parra‐Damas et al., 2017). BDNF, the downstream target gene of CREB, has also been shown to be regulated by stress, antidepressants, and antipsychotics (Jin et al., 2017; Liao et al., 2017; Xue et al., 2016).

**FIGURE 1:**
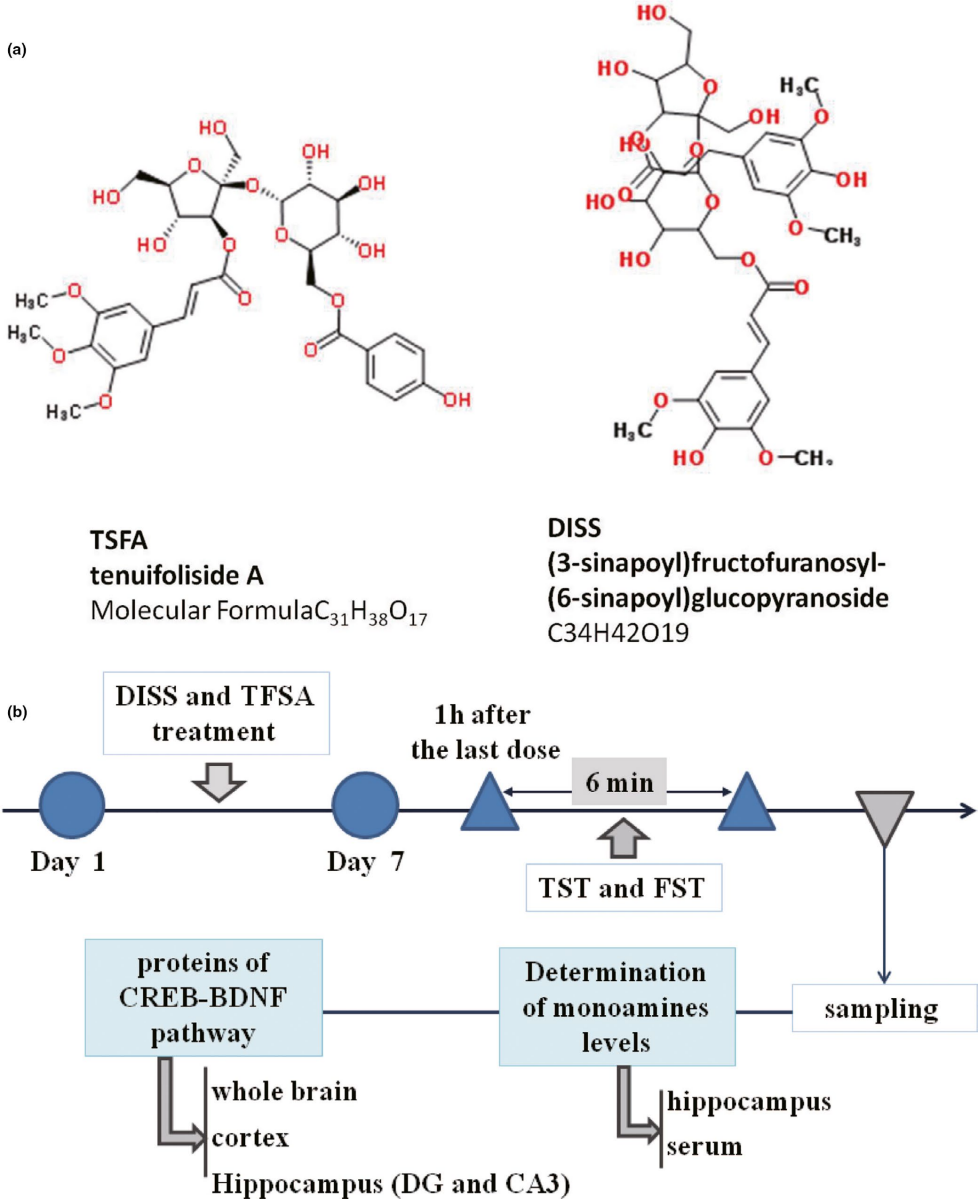
The structure of TFSA and DISS and flow chart

Importantly, herbal products have been widely explored as supplements especially in the traditional Chinese medicine (TCM), where they exclusively play an important role in dealing with various diseases (Ma et al., 2009). Especially from the past, the extract of Radix Polygalae (the root of wild *Polygala tenuifolia*) has been used to treat mental disorders as tranquillizer, antidepressant, and antipsychotic agent (Cao et al., 2016; Hu et al., 2009; Hu, Liu, et al., 2010). The main active principles/components of this extract are 3,6'‐Disinapoyl sucrose (DISS) and Tenuifoliside A (TFSA), whose chemical structures have been depicted in Figure 1a. Earlier studies have demonstrated that DISS and TFSA both possess neuroprotective and antidepressant properties (Hu et al., 2011, 2012; Hu, Liao, et al., 2010). In addition, studies have also revealed that both these compounds target downstream genes through different mechanisms. TFSA typically promotes neurite outgrowth in PC12 cells via regulating Phosphoinositide 3‐kinase (PI3K)/ Protein Kinase B (AKT) and ERK kinase (MEK)/ extracellular‐signal‐regulated kinase (ERK)/CREB signaling pathways (Liu et al., 2016), while neuroprotective effects of DISS involved regulation of Calcium/calmodulin‐dependent protein kinase type II (CaMKII)/CREB and ERK1/2/CREB pathways (Hu et al., 2014). These findings led us to hypothesize that coadministration of both these compounds might stimulate the protein expression and phosphorylation of CREB and its downstream signaling pathway (Dong et al., 2014; Hu, Liao, et al., 2010).

## 2 METHODS

### 2.1 Materials

5‐hydroxytryptamine (5‐HT) was purchased from Sigma Chemical Co. (USA, Cat: H9523). The DISS and TSFA were isolated from *Polygala tenuifolia*, identified by a combination of spectra methods (UV, IR, MS, and NMR), and purified through high‐performance liquid chromatography (HPLC) (purity > 90%). *Polygala tenuifolia* was purchased from the LvYe Medicinal Material Company, China, and identified by Professor Ping Liu (Pharmacy Care Center, Chinese PLA General Hospital). The quality of the crude drug is controlled by the Chinese Pharmacopoeia (2010). The voucher specimen of plant was registered under the number NU‐80617 and deposited in the Herbarium of Traditional Chinese Medicinal pharmacy, Chinese PLA General Hospital, China. Fluoxetine was obtained from Eli Lilly (Indianapolis, USA). The rabbit polyclonal antibodies directed against BDNF (Cat: Abcam ab108319), CREB ( Cat: CST #9197), phospho‐CREB ( Ser133, Cat: CST #9198), and α‐tubulin ( Cat: CST #2125) proteins were obtained from Cell Signaling Technology (Beverly, MA, USA) or Abcam (CA, USA). The horseradish peroxidase (HRP)‐conjugated secondary antibodies were procured from Santa Cruz Biotechnology (Santa Cruz, CA, USA).

### 2.2 Animals and drug treatment

Male KM mice (18–22 g) were purchased from Vital River Laboratory Animal Technology Co. (Beijing, China) and housed in a 12‐hr light: dark cycle, at a constant temperature (22 ± 2°C) in groups of five in each cage, with free access to food and water. The experimental protocol (Figure 1b) was approved by the Committee on the Ethics of Animal Experiments of Chinese PLA General Hospital (Permit Number: 20160307‐049). The mice were divided into 10 different groups, with 8–12 mice in each group, and were administered with saline (control); Fluoxetine 20 mg/kg; saline + DISS 5 mg/kg; saline + TSFA 5 mg/kg; saline + DISS 10 mg/kg; saline + TSFA 10 mg/kg; DISS 5 mg/kg + TSFA 5 mg/kg; DISS 5 mg/kg + TSFA 10 mg/kg; DISS 10 mg/kg + TSFA 5 mg/kg; DISS 10 mg/kg + TSFA 10 mg/kg, intraperitoneally for 7 days, forced swimming or tail suspension test were started 60 min after the last dose. All the compounds were dissolved in saline immediately, before the injections. In terms of coadministration of two drugs, they were injected immediately one after another. Animals were decapitated, and specimens were collected 24 hr after the end of last treatment.

### 2.3 Forced Swimming Test (FST) and Tail Suspension Test (TST)

These experiments involving FST and TST were carried out in mice according to the method described by Hufgard et al. (2017). Briefly in FST, on day 1, mice were placed in the glass cylinder measuring 20 cm with a water up to 10 cm high and at 25 ± 1°C temperature for 15 min. On day 2, mice were given a second trial for 6 min and scored for immobility. However, for TST, each mice were individually suspended by its tail using a clamp (2 cm from the end) for 6 min in a box (25 × 25 × 30 cm) with the head 5 cm from the bottom. The testing was carried out in a darkened room with minimal background noise. The duration of immobility was recorded during the 4‐min (3–6 min) test period.

### 2.4 Determination of monoamines levels

After the mice decapitation, their brain regions (frontal cortex and hippocampus) were rapidly removed and placed on ice. The removed brain tissues were weighed and stored at −80°C, until homogenization. Later, the protein concentration of the brain tissue was calculated using BCA assay, and subsequently, the levels of serotonin, noradrenaline, and dopamine were measured by HPLC. Briefly, after homogenization of the frozen tissue samples by ultrasonication, the tissue extraction solution (solution A, 1.43 mM EDTA‐Na_2_, 8.25 mM l‐cysteine, and 0.14 mM perchloric acid) was added at the following ratio of 4 μl/mg. After this, homogenate was kept on ice for 20 min, and later, 2 volumes (of the tissue weight) of solution B, including 90 mM sodium dihydrogen phosphate, was added. After the incubation of the mixture on ice for 1 hr, the samples were centrifuged at 20627 g (4°C) for 15 min. The 50 μl volume of the resultant supernatant was directly added to the HPLC system equipped with reversed‐phase C18 column and an electrochemical detector (ESA CoulArray, Chelmstord, MA, USA). The mobile phase consisted of 30 mM sodium dihydrogen phosphate, 1 mM sodium heptane sulfonic acid, and 15% methanol (pH = 3.06), and the flow rate was 1 ml/min. Finally, the tissue levels of monoamine were expressed in terms of nanograms per gram of tissue.

### 2.5 Tissue collection and Western blotting

The removed whole brains from the FST mice were used for further dissections, which were performed on a glass plate with cold 0.01 M phosphate‐buffered saline (PBS). All the collected tissues were quickly frozen in liquid nitrogen and preserved at −80°C. The immunoblotting was performed as described previously (Hu et al., 2014). The protein separated on membranes after electrophoresis was probed overnight with primary monoclonal antibodies (mAbs), α‐tubulin was used as an internal control and followed by detection with HRP‐conjugated secondary Abs. The final signal was quantified by measuring the optical density using an image analysis system (Bio‐Imaging Analyzer, UVP). Relative protein expression was further quantified using ImageJ 1.29 software (National Institutes of Health, Bethesda, MD).

### 2.6 Immunohistochemical Analysis

The brain tissue sections from four mice were randomly selected from each group, and the hippocampus and frontal cortex were removed, before immersing them in 4% paraformaldehyde for 24 hr. Next, the coronal brain slices, about 3 mm thick, were cut after dissecting 7 mm frontal pole (16 µm) and later embedded into the paraffin blocks. Standard histological processing was performed using these paraffin‐embedded sections. Briefly, the endogenous peroxidase activity was blocked by immersing these sections in methanol containing 0.3% hydrogen peroxide for 15 min and later washing with PBS. Next, after adding the antigen retrieval solution, the sections were incubated in the microwave oven, at medium high setting for 6~10 min. After cooling of the sections to room temperature post antigen retrieval, normal non immune animal serum (goat serum) was added and was incubated for 15 min at 37°C. Thereafter, the serial sections were separately incubated overnight at 4°C with primary antibodies for CRTC1 (1:200), pCREB (1:100), CREB (1:100), and BDNF (1:200; rabbit anti‐rat antibody, Abcam, USA). Next day, after washing with PBS, the sections were further incubated at room temperature with anti‐rabbit secondary antibody for 30 min. Finally, the staining signal for BDNF, pCREB, CREB, and CRTC1 proteins was visualized by incubating the section with 3, 3‐diaminobenzidine tetrahydrochloride for 4 min. Subsequently, the stained sections were photographed using a light microscope (LeicaDMI4000B, Germany). Immunopositive cells in the cortex, CA3, and DG regions from five sections (the thickness of paraffin section is 4 μm, take the first piece every five consecutive sections) per mice were counted using an image analysis system (Microsystems AG, Wetzlar, Germany). The integrated optical density (IOD, optical density per unit area) reflects the number of positive cells.

### 2.7 Statistical analysis

All data were analyzed using GraphPad Prism software version 5 (Graph Pad software Inc., San Diego, CA, USA) and were presented as mean ± *SEM* in three independent experiments, where each experiment was performed in triplicate. And mean ± *SD* was used for behavioral statistics. Data were grouped according to the treatment and analyzed using analysis of variance (ANOVA) followed by Tukey's post hoc test. The *p* value of <.05 was considered to represent statistically significant difference.

## 3 RESULTS

### 3.1 DISS and TFSA combined treatment showed synergistic antidepressant‐like effect

**FIGURE 2:**
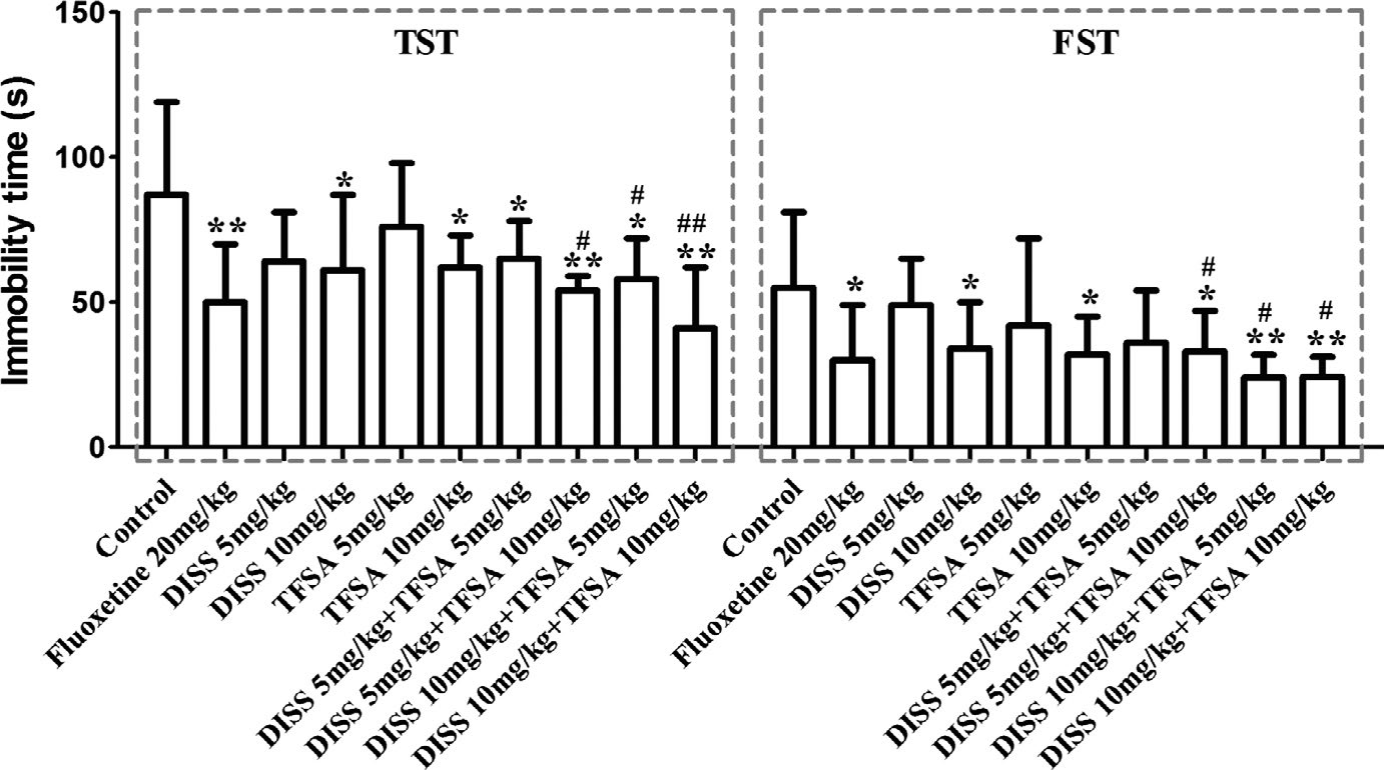
Analysis of the effects of DISS and TFSA administration on the immobility time of mice by tail suspension test (TST) and forced swimming test (FST). DISS and TFSA were administered at indicated dose combinations, and 60 min later, the FST and TST were performed (*n* = 10–12/group). *p < .05, **p < .01, compared with control; ^#^ *p* < .05, ^##^ *p* < .01, compared with the DISS (10 mg/kg)

As observed in Figure 2, the combined treatment of DISS and TFSA at a dose of 10 mg/kg each reduced mice immobility and induced antidepressant‐like activity. We tested four combinations of these two compounds in mice using variable doses. In fact, subactive (5 mg/kg) or active doses (10 mg/kg) of DISS and TFSA led to significant reduction of mouse immobility in all combinations, and the effects were more significant when they were coadministrated, in comparison with their single dose administration in both models of despair test (*p* < .05). The immobility in the combined active dose of DISS and TFSA (10 mg/kg each) group displayed the most inhibition (inhibition ratio in TST was 52.60%; *p* < .01).

### 3.2 Analysis of the effects of combined DISS and TFSA treatment on monoamines levels

**FIGURE 3:**
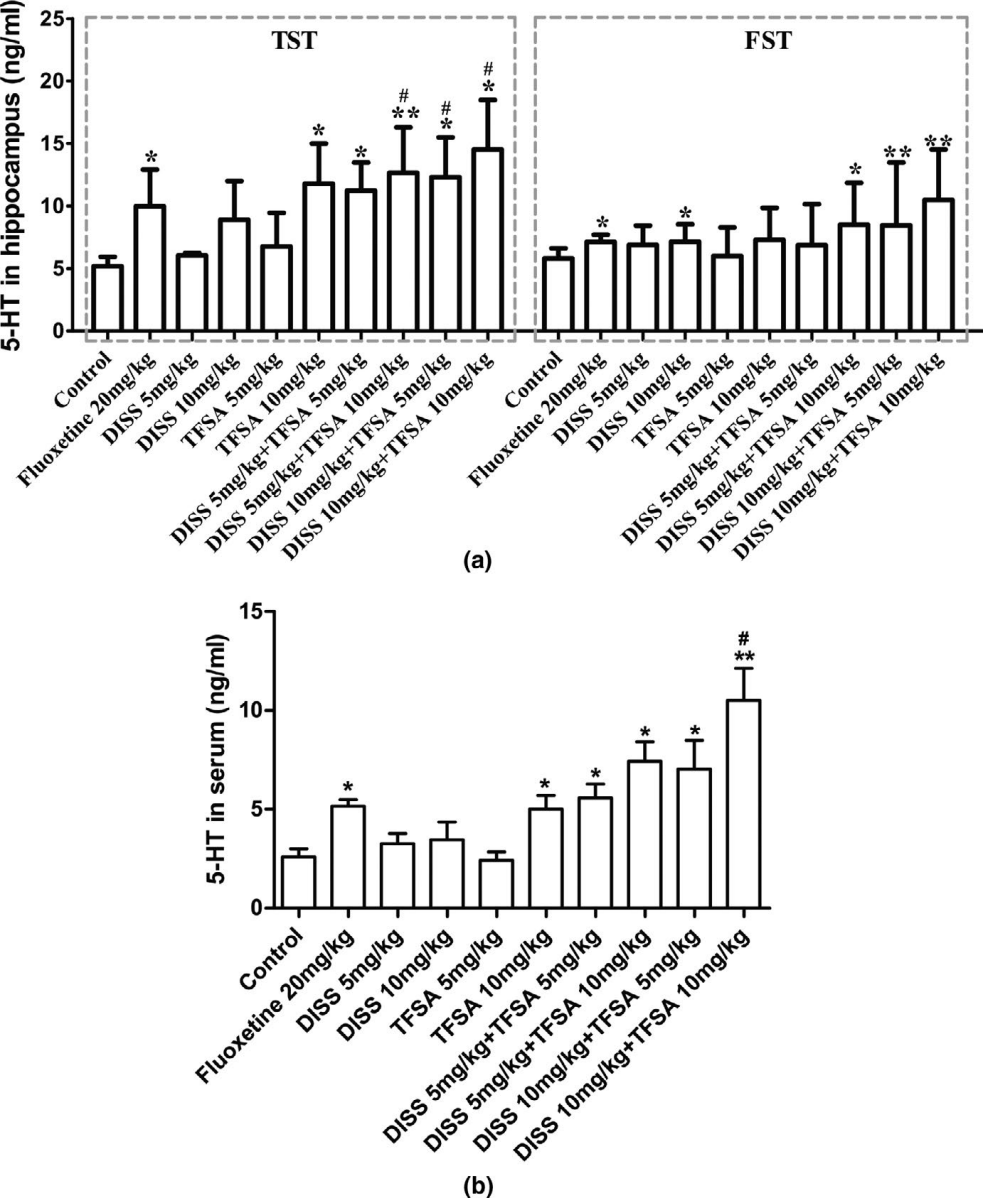
Influence of DISS and TFSA on the levels of 5‐HT in the hippocampus during TST and FST and the levels of 5‐HT in the serum. (a) 5‐HT in the hippocampus in TST and FST mice; (b) 5‐HT in the serum in FST mice. *p < .05, **p < .01, compared with control; ^#^ *p* < .05,^##^ *p* < .01, compared with the DISS (10 mg/kg) or TFSA (10 mg/kg), (*n* = 10–12/group)

The levels of 5‐HT in the hippocampus and serum after treatment with DISS and TFSA have been shown in Figure 3. No increase in 5‐HT levels was noticed at either DISS or TFSA single treatment, except DISS treatment at 10 mg/kg in FST. However, combined treatment of DISS and TFSA, at a concentration of 10 mg/kg each, showed increasing trend of 5‐HT levels both during TST and FST. In addition, during TST, increased 5‐HT levels were observed in all combination groups, in comparison with DISS (10 mg/kg) alone (*p* < .05). There was no significant change in NE and DA levels after treatment (results not shown).

### 3.3 DISS and TFSA combined treatment showed synergistic effect on key proteins of CREB‐BDNF pathway

**FIGURE 4:**
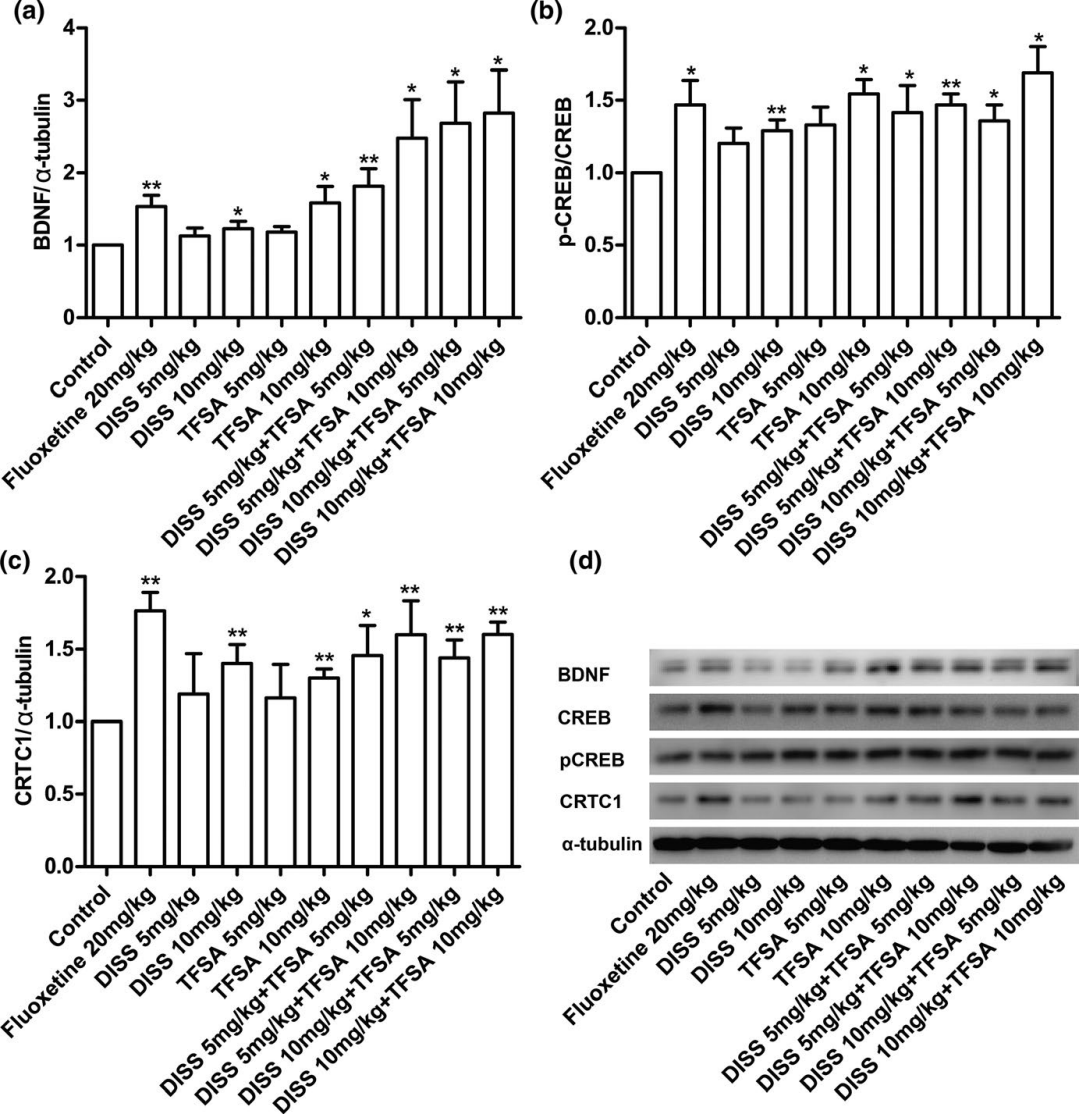
Effects of DISS and TFSA on the protein expression of CRTC1, BDNF, and pCREB in brain. (a) A representative image of BDNF, CREB, p‐CREB, CRTC1, and α‐tubulin expression in different groups, α‐tubulin was used as an internal control; (b) The ratio of BDNF to α‐tubulin; (c) The ratio of p‐CREB/CREB; (d) The ratio of CRTC1 to α‐tubulin; the ratio in the control group was set as 1. *p < .05; **p < .01

Furthermore, we also examined the effect of DISS and TFSA combined treatment on CREB‐CRTC1‐BDNF signaling pathway, by analyzing the induction of CRTC1, pCREB,, and BDNF in the whole brain through Western blotting (Figure 4a–d). The DISS and TFSA at the dose of 10 mg/kg each showed maximum significant increase in CRTC1 (*p* < .01), BDNF (*p* < .05), and phosphorylated CREB (*p* < .05) protein levels. In addition, all other combinations of DISS and TFSA treatment also showed some increase in CRTC1, BDNF, and phosphorylated CREB protein levels, in comparison with positive control. However, the best synergistic effect on pCREB, CTRC1, and BDNF levels was observed with combined DISS and TFSA treatment, both at a dose of 10 mg/kg each (*p* < .05; Figure 4).

**FIGURE 5:**
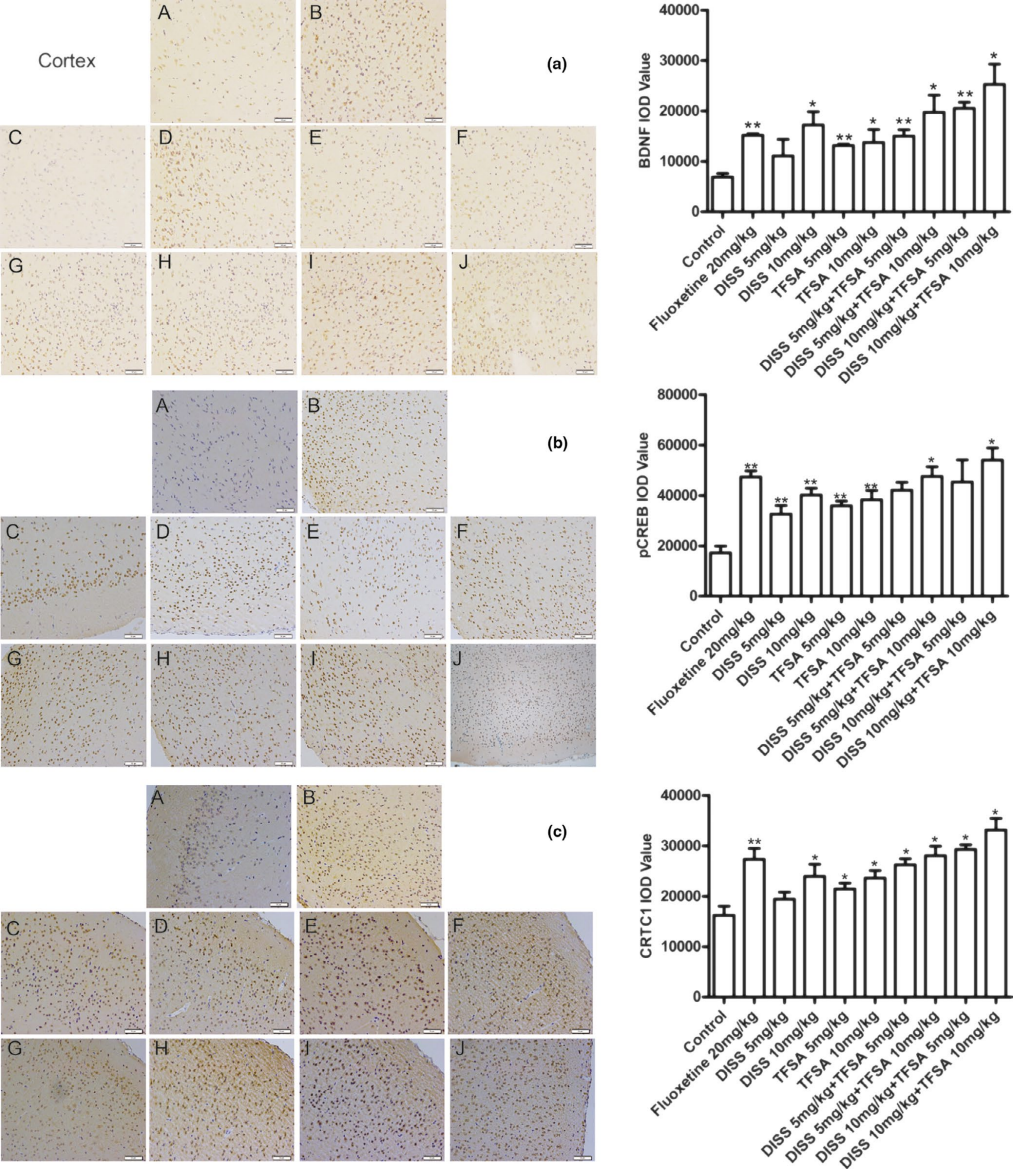
Effects of DISS and TFSA on the expression of CRTC1, BDNF, and pCREB through IHC in cortex of FST mice, under 200× magnification. A: Control group; B: Fluoxetine group; C: DISS 5 mg/kg; D: DISS 10 mg/kg; E: TFSA 5 mg/kg; F: TFSA 10 mg/kg; G: DISS 5 mg/kg + TFSA 5 mg/kg; H: DISS 5 mg/kg + TFSA 10 mg/kg; I: DISS 10 mg/kg + TFSA 5 mg/kg; J: DISS 10 mg/kg + TFSA 10 mg/kg. *p < .05, **p < .01, versus the control group

Similarly, through immunohistochemical analysis, we independently verified that only combined treatment of DISS and TFSA provided an obvious synergistic aggrandize CTRC1, pCREB, CREB, and BDNF immunopositive cells. In addition, except for separate DISS (5 mg/kg) and TFSA (5 mg/kg) treatments, all other treatment groups showed significant increase in the immunopositive CTRC1 cells in the cortex (*p* < .05), in comparison with control group, and these effects seem to be synergistic (Figure 5c). Also, similar increasing trend was observed in the case of pCREB immunopositive cells in the cortex with most of the combination treatments (*p* < .05; Figure 5b). In the case of BDNF, all treatment groups increased BDNF immunopositive cells in the cortex, but some combination groups like DISS 5 mg/kg plus TFSA 10 mg/kg, and DISS 10 mg/kg plus TFSA 5 mg/kg (*p* < .01), produced stronger effects in comparison with fluoxetine (Figure 5a).

**FIGURE 6:**
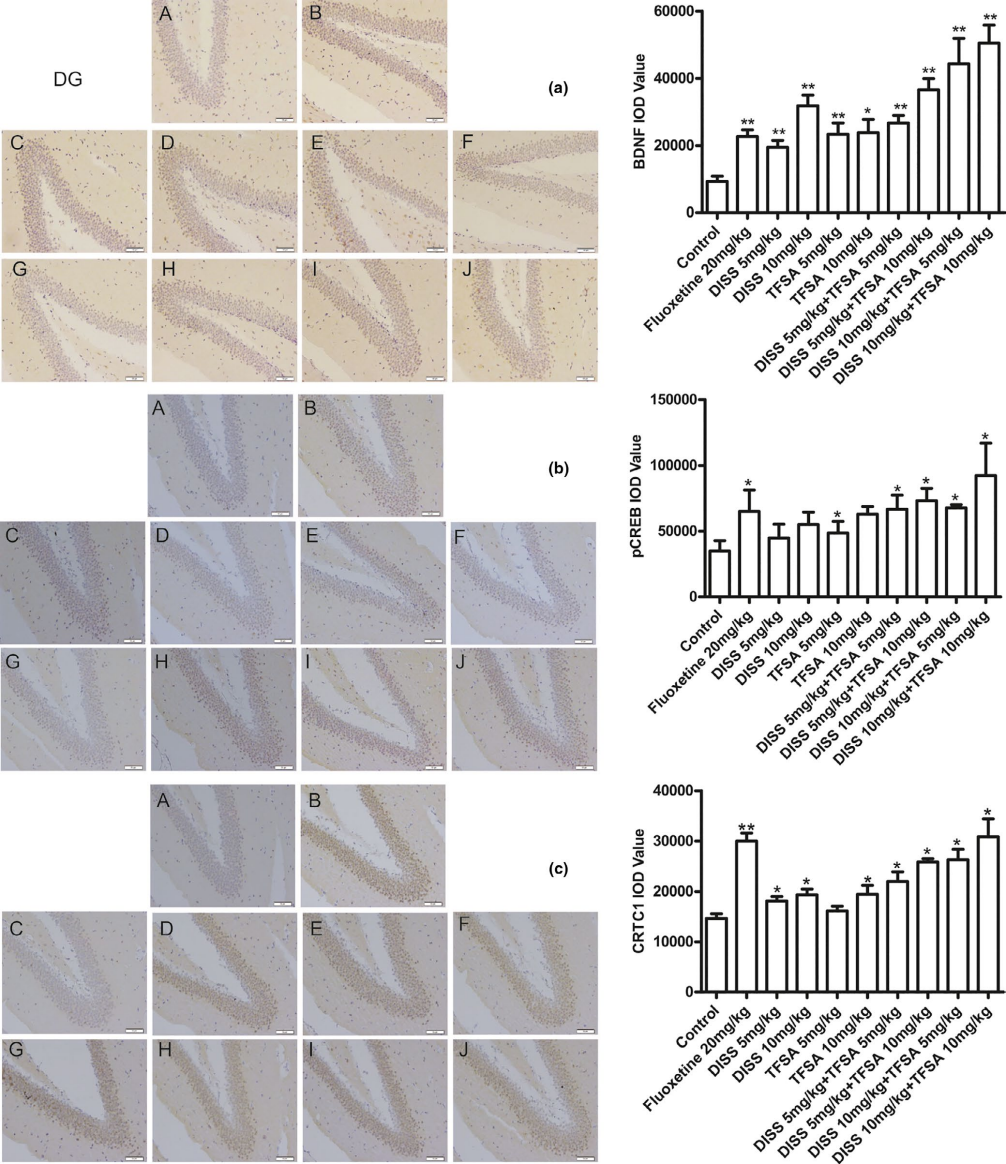
Effects of DISS and TFSA on the expression of CRTC1, BDNF, and pCREB through IHC in hippocampus DG areas of FST mice, under 200× magnification. A: Control group; B: Fluoxetine group; C: DISS 5 mg/kg; D: DISS 10 mg/kg; E: TFSA 5 mg/kg; F: TFSA 10 mg/kg; G: DISS 5 mg/kg + TFSA 5 mg/kg; H: DISS 5 mg/kg + TFSA 10 mg/kg; I: DISS 10 mg/kg + TFSA 5 mg/kg; J: DISS 10 mg/kg + TFSA 10 mg/kg. *p < .05, **p < .01, versus the control group

**FIGURE 7:**
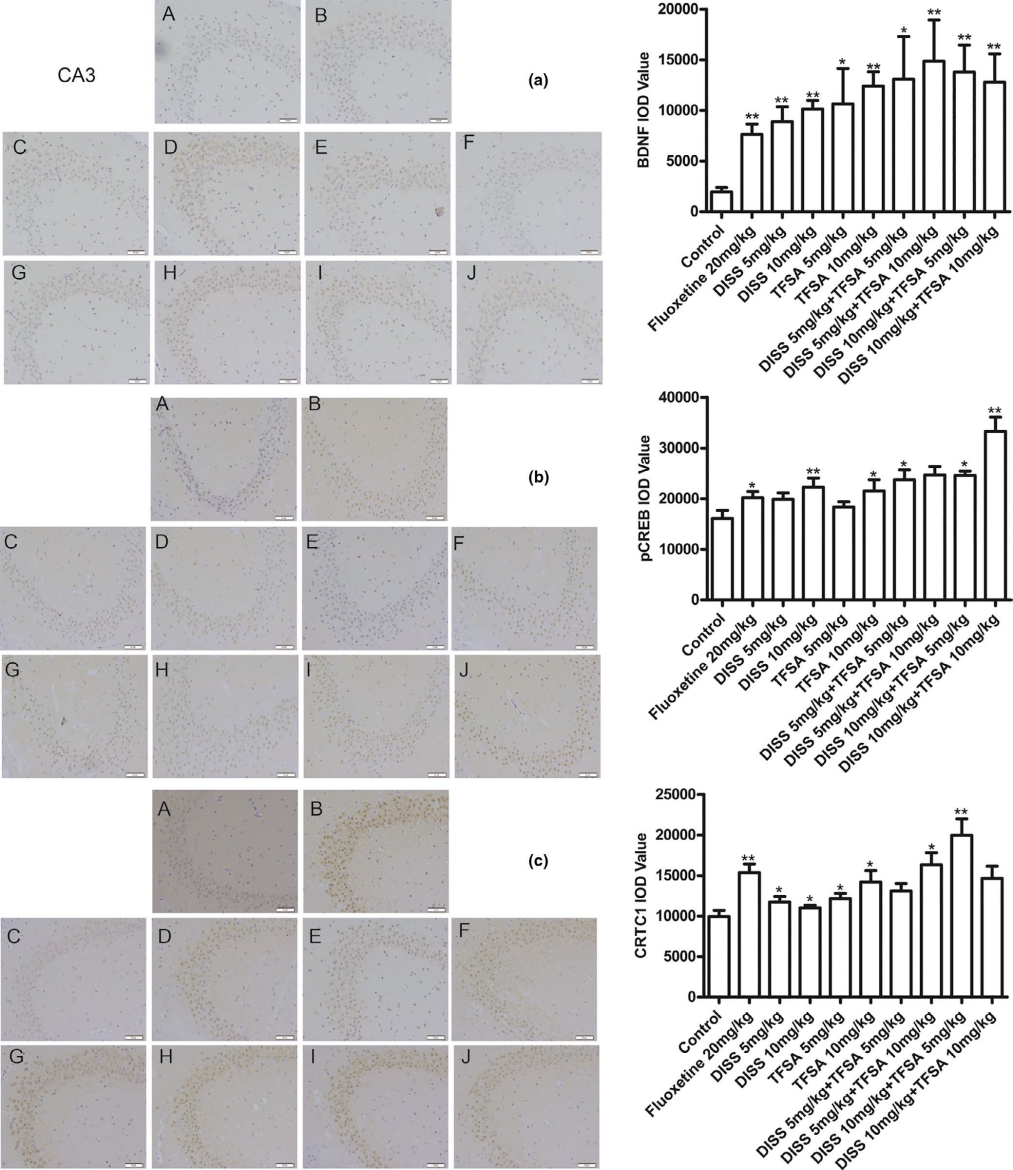
Effects of DISS and TFSA on the expression of CRTC1, BDNF, and pCREB through IHC in hippocampus CA3 areas of FST mice, under 200× magnification. A: Control group; B: Fluoxetine group; C: DISS 5 mg/kg; D: DISS 10 mg/kg; E: TFSA 5 mg/kg; F: TFSA 10 mg/kg; G: DISS 5 mg/kg + TFSA 5 mg/kg; H: DISS 5 mg/kg + TFSA 10 mg/kg; I: DISS 10 mg/kg + TFSA 5 mg/kg; J: DISS 10 mg/kg + TFSA 10 mg/kg. *p < .05, **p < .01, versus the control group

Except for separate DISS (5 mg/kg) and TFSA (5 mg/kg) treatments, all other treatment groups showed significant increase of in the immunopositive CTRC1, Pcreb, and BDNF cells in the DG areas (Figure 6) and CA3 areas (Figure 7) of hippocampus, in comparison with control group. In DG areas, DISS (10 mg/kg) combined TFSA (10 mg/kg) showed the strongest regulatory effect on pCREB (*p* < .05), CTRC1 (*p* < .05), and BDNF (*p* < .01) levels. However, the BDNF expression in the CA3 area was only increased with DISS plus TFSA, but no synergistic effect was observed and could be due to high standard deviation (Figure 7a).

## 4 DISCUSSION

Multiple studies have suggested that combination therapy offers some potential advantages as antidepressants including less withdrawal syndrome and more rapid or effective clinical response (Li et al., 2015; McAllister‐Williams et al., 2013). There have also been reports indicating that total content of the herbal product correlate with significantly better synergistic effect than an equivalent dose of a single isolated active ingredient in MDD (Leonard et al., 2002; Williamson, 2001). Even herbal combinations have been observed to be more effective than the constituent herb used as alone (Scholey & Kennedy, 2002). Consistent with these reports, our study also revealed that administration of two oligosaccharide esters (DISS and TFSA) in combination displayed (a) synergistic antidepressive effects and definitely exceeded those obtained by either compound used alone in FST and TST; (b) increased 5‐HT levels in the hippocampus; and (c) activated the related CREB‐CRTC1‐BDNF cascade pathway.

Previous similar behavioral study suggested that intake of natural DISS (best effective dosage 20 mg/kg) induced significant increase in sucrose intake under chronic mild stress (CMS) in rats (Hu, Liao, et al., 2010; Hu, Liao, Liu, Guo, & Rahman, 2009). Likewise, TFSA have also been shown to possess antidepressant‐like effects (Dong et al., 2014). The mouse TST and FST are widely used rodent models of behavioral despair in the study of depression (Chen et al., 2018). This typical “immobile state” of animals reflects a so‐called “behavioral despair state.” This behavioral despair model is similar to depression and sensitive to most antidepressants, and its efficacy is significantly related to clinical efficacy. In our study, DISS and TFSA both showed anti‐immobility response, but the effect was not obvious when subactive dosage was administered. However, the positive effects were again observed when mice were treated with DISS (10 mg/kg) and subthreshold dose of TFSA, or other way round. The combined DISS and TFSA treatment at high dosage even demonstrated greater improvement in anti‐immobility than individual treatment.

Mounting numbers of studies have demonstrated that monoamine neurotransmitters, particularly noradrenaline and 5‐HT, in the brain are reduced in depressed patients and in preclinical animal models with depressive‐like behaviors (Blier, 2013). In our study, we observed that DISS and TFSA synergistically increased monoamine transmitters, including 5‐HT in the hippocampus region, which is highly relevant to emotional disorders. Neurotransmitters are the other factors thought to regulate adult neurogenesis (Berg et al., 2013). Especially, 5‐HT has long been considered as a positive regulator of neurogenesis (Banasr et al., 2004).

The prefrontal cortex is involved in the regulation of mood and stress, and changes in the expression of BDNF and 5‐HT in the prefrontal cortex have been implicated in the pathophysiology of depression and the therapeutic effect of antidepressants (Zhou et al., 2016). The common feature between 5‐HT and BDNF is their ability to regulate development and plasticity of neural circuits, involved in mood disorders like depression and anxiety. Specifically, BDNF promotes the survival and differentiation of 5‐HT in the neurons. Conversely, administration of antidepressant selective serotonin reuptake inhibitors (SSRIs) enhances BDNF gene expression (Udina et al., 2016). Importantly, it is presumed that neurotransmitters contribute to neurogenesis through the stimulation of receptors on postsynaptic neurons, with effects on intracellular secondary messengers and protein kinases, which leads to enhanced expression of BDNF, and subsequently promotion of neurogenesis (Willner et al., 2013). It has been shown that increase in the extracellular levels of 5‐HT in turn increases BDNF levels by inhibiting serotonin transporter (5‐HTT) through 5‐HT1 receptor subtypes and is positively coupled with adenylate cyclase and protein kinase A (PKA) (Kim & Leem, 2014; Udina et al., 2016). This then results in increased 5‐HT1AR protein levels and activation of 5‐HT1A receptor (5‐HT1AR)‐mediated cAMP‐PKA‐CREB signaling pathway in hippocampal neurons (Wang et al., 2018).

In the past decades, BDNF, a member of the neurotrophin family, has been documented to play essential roles in neurotrophic support during neurodevelopment and neurogenesis. In addition, previous studies have also demonstrated the role of CREB signaling pathway and CRTC1‐related CREB transcription in modulating BDNF expression (Breuillaud et al., 2012; Parra‐Damas et al., 2017). The in vitro studies have revealed that DISS regulated not only BDNF expression through CREB‐mediated transcription, but also upstream activation of ERK1/2 and CaMKII in its neuroprotective effects (Hu, Liao, et al., 2010; Hu et al., 2011). In a separate study, the mechanism of TFSA function was found to be related to TrkB/BDNF/ERK and TrkB/BDNF/PI3K signaling pathways in C6 cells (Dong et al., 2014). However, in our study, we demonstrated that DISS and TFSA exerted additive neuroprotective effects on CREB‐BDNF signaling pathway, through complementary mechanism (X. Liu et al., 2016). Moreover, our results indicated that combined treatment of DISS and TFSA was more powerful in increasing pCREB, CRTC1, and BDNF levels, in comparison with administration of DISS and TSFA alone. Thus, our study demonstrated that 5‐HT and its downstream CREB –BDNF signaling pathway may be responsible for the antidepressive effects of combined DISS and TFSA treatment.

## 5 CONCLUSIONS

Overall, we identified that combined treatment of DISS and TFSA exerts superior synergistic antidepressant therapeutic effects in despair mice model, as characterized by decrease in immobility behavior through FST and TST. Additionally, the synergistic mechanism might be mediated through 5‐HT/CREB‐CRTC1‐BDNF pathway. Therefore, we propose that combined administration of two oligosaccharide esters has great potential to be developed as neuroprotective herbal remedy against depressive‐like impairment and relevant neuropsychiatric disorders.

## CONFLICT OF INTEREST

The authors declare that they do not have any conflict of interest.

## ETHICAL REVIEW

This study was approved by the Committee on the Ethics of Animal Experiments of Chinese PLA General Hospital (Permit Number: 20160307‐049).

## INFORMED CONSENT

Written informed consent was obtained from all study participants.

## Data Availability

The datasets used and/or analysed during the current study are available from the corresponding author on reasonable request.
